# Expression in the human brain of retinoic acid induced 1, a protein associated with neurobehavioural disorders

**DOI:** 10.1007/s00429-014-0712-1

**Published:** 2014-02-12

**Authors:** Yara Dadalti Fragoso, Patrick N. Stoney, Kirsty D. Shearer, Angelo Sementilli, Sonia E. Nanescu, Pietro Sementilli, Peter McCaffery

**Affiliations:** 1 Department of Neurology, Universidade Metropolitana de Santos, Santos, SP Brazil; 2 Institute of Medical Sciences, University of Aberdeen, School of Medical Sciences, Foresterhill, Aberdeen, AB25 2ZD Scotland, UK; 3 Department of Physiopathology, Universidade Metropolitana de Santos, Santos, SP Brazil

**Keywords:** Transcription, Cytoplasmic, Smith–Magenis, Potocki–Lupski, Retinoic acid, RAR, Cerebellum, Hippocampus, Cerebral cortex

## Abstract

Retinoic acid induced 1 (RAI1) is a protein of uncertain mechanism of action which nevertheless has been the focus of attention because it is a major contributing factor in several human developmental disorders including Smith–Magenis and Potocki–Lupski syndromes. Further, RAI1 may be linked to adult neural disorders with developmental origins such as schizophrenia and autism. The protein has been extensively examined in the rodent but very little is known about its distribution in the human central nervous system. This study demonstrated the presence of *RAI1* transcript in multiple regions of the human brain. The cellular expression of RAI1 protein in the human brain was found to be similar to that described in the mouse, with high levels in neurons, but not glia, of the dentate gyrus and cornus ammonis of the hippocampus. In the cerebellum, a second region of high expression, RAI1 was present in Purkinje cells, but not granule cells. RAI1 was also found in neurons of the occipital cortex. The expression of this retinoic acid-induced protein matched well in the hippocampus with expression of the retinoic acid receptors. The subcellular distribution of human neuronal RAI1 indicated its presence in both cytoplasm and nucleus. Overall, human RAI1 protein was found to be a highly expressed neuronal protein whose distribution matches well with its role in cognitive and motor skills.

## Introduction

The *retinoic acid induced 1* (*RAI1*) gene is highly conserved through mammalian evolution (Girirajan et al. 2005), while the corresponding protein is known to be expressed at high levels in the heart and neuronal structures (Toulouse et al. 2003). Its precise action is unclear, but from its similarity to the transcriptional regulator TCF20 (also called stromelysin-1 platelet-derived growth factor-responsive element binding protein, SPBP), it may be part of a complex that regulates transcription and indeed it can act as a transactivator (Seranski et al. 2001; Bi et al. 2004; Carmona-Mora et al. 2010). A lot of interest surrounds RAI1 because of its involvement in neurobehavioral disorders in an intriguing dosage-sensitive manner (Carmona-Mora and Walz 2010). Haploinsufficiency of the *RAI1* gene is associated with Smith–Magenis syndrome, a rare disorder that includes craniofacial, behavioural and neurological signs including intellectual difficulties and sleep disturbance, as well as obesity (Slager et al. 2003; Girirajan et al. 2005). Many features of Smith–Magenis syndrome, originally described as resulting from an interstitial deletion in chromosome 17p11.2 (Smith et al. 1986), are caused by the loss of *RAI1* which is located in this chromosomal region. In contrast, duplication of this same chromosomal region results in another rare disorder, Potocki–Lupski syndrome, whose features also include neurobehavioral difficulties including features of autism, hypotonia and cardiovascular anomalies. Again, the change in expression in RAI1 is proposed to contribute to the disorder (Cao et al. 2013). RAI1 is also associated with neurodevelopmental disorders that are pervasive into adulthood including schizophrenia (Toulouse et al. 2003) and autism (Carmona-Mora and Walz 2010) as well as adult diseases such as Parkinson’s disease (Do et al. 2011) and cerebellar ataxia (Hayes et al. 2000). The potential action of RAI1 as a regulator of transcription is thus a key to normal function of the adult brain.

*RAI1* was first described as a retinoic acid-regulated gene (Imai et al. 1995) from whence derived its name. Retinoic acid is the bioactive metabolite of vitamin A, acting through the ligand-gated nuclear receptor RAR, and which influences motor function, memory and behaviour (Shearer et al. 2012). The initial discovery of *RAI1* came from study of the P19 mouse embryonic carcinoma cell line, showing that the expression of *GT1*, a splice variant of *RAI1*, was significantly up-regulated after treatment with retinoic acid to induce neuronal differentiation (Imai et al. 1995). The *RAI1* promoter contains multiple retinoic acid response elements (RAREs) in its promoter (Laperriere et al. 2007) although there has been no description since that time of regulation of *RAI1* by retinoic acid.

Despite the growing knowledge of the role of RAI1 in human neurological and psychiatric diseases, and its high expression in brain (Toulouse et al. 2003), the distribution of this protein in the human brain has yet to be described. The present study aimed to investigate the expression of RAI1 in human hippocampus, cortex and cerebellum, areas likely involved in cognitive and motor functions of RAI1 neural expression (Elsea and Girirajan 2008). In these regions, RAI1 was expressed in neurons, but not GFAP-positive glia. The subcellular distribution of endogenous RAI1 implied both nuclear and cytoplasmic localization, differing from what has been described when RAI1 is overexpressed in cell lines.

## Methods

### Tissue samples

The present study was approved by the Ethics Committee of Universidade Metropolitana de Santos, SP, Brazil, and by the Brazilian Health Research Committee on April 4th 2011, designated CONEP 16168, documents registered as 25000.169694/2010-18. Caudal human hippocampi, cerebellar and cerebral cortices were obtained from six male individuals aged 55 years or less who did not present any neurological or psychiatric disease and were collected during necropsy procedures. Brains from individuals whose death was related to head trauma, extensive infection or toxic, anoxic or metabolic injuries were excluded from this study. For immunohistochemistry, samples from the hippocampus, cerebral cortex and cerebellum, measuring typically 0.5 cm^3^, were fixed in 10 % phosphate-buffered formalin within 24 h of death and processed into paraffin wax blocks within the following 24 h. Further samples from the same areas were collected in RNAlater RNA Stabilization Reagent (Qiagen, Venlo, The Netherlands) and stored at 4 °C for qPCR.

### Immunohistochemistry

Fluorescence immunohistochemistry was performed as previously described in (Fragoso et al. 2012), adapted from an earlier study (Makitie et al. 2010). Wax-embedded tissue samples were sectioned at 7 μm, mounted onto A380-bond slides (Electron Microscopy Sciences, Hatfield PA 19440, USA) and dried overnight at 37 °C. Mounted sections were dewaxed in xylene and rehydrated through decreasing ethanol concentrations (100, 95, 80 and 70 %). Sections were then boiled for 10 min in sodium citrate buffer, pH 6.0, allowed to cool on the bench for 20 min and then washed in phosphate-buffered saline (PBS) pH 7.4, containing 0.1 % Tween 20 (Sigma) and 1 % pooled human serum (BioSera). Sections were then blocked for 1 h at room temperature in PBS containing 0.3 % Tween 20, 5 % normal goat serum, 5 % bovine serum albumin and 5 % pooled human serum. The sections were next incubated overnight at 4 °C in primary antibody diluted in blocking solution. The following primary antibodies were used: mouse anti-Calbindin (1:500; Sigma, C9848), mouse anti-GFAP (1:500; Sigma, G3893), chicken anti-MAP2 (1:1000; Abcam, ab5392), mouse anti-RAI1 (1:500; Santa Cruz, D-11, sc-365065), rabbit anti-RAI1 (1:500; Abcam, ab58658). After incubation, the slides were washed in blocking solution, then incubated with a fluorescent secondary antibody (anti-rabbit 1:300, Invitrogen or anti chicken 1:400, Jackson Immunoresearch) diluted in blocking solution for 1 h at room temperature. Slides were washed in blocking solution and incubated for 1 min with 10 % Sudan Black (Acros Organics) in 70 % isopropanol to reduce autofluorescence (Schnell et al. 1999; Neumann and Gabel 2002). The slides were then thoroughly washed in distilled water and mounted with mounting medium containing 1,4-diazabicyclo[2.2.2]octane (DABCO, Sigma) and bisbenzimide (Sigma). The sections were digitally imaged on a Zeiss Axio Imager M2 or a Zeiss LSM710 confocal microscope on an inverted Axio Observer Z1 stand.

### Quantitative polymerase chain reaction (qPCR)

qPCR was carried out as previously described (Fragoso et al. 2012). Total RNA was extracted from tissue samples using an Isolate RNA kit (Bioline) and cDNA synthesized using a High Capacity RNA-to-cDNA kit (Applied Biosystems). qPCR was carried out using SensiMix SYBR qPCR master mix (Bioline) and primers designed to amplify human *RAI1*: forward: CCC AGG AGC ACT GGG TGC ATG A, reverse: GCA GCT GGA ACA CAT CAT GTC CAC G. The reference gene used was *GAPDH*, forward: TCT TTT GCG TCG CCA GCC GA, reverse: AGT TAA AAG CAG CCC TGG TGA CCA. Standard curves and blank controls were run for both genes. Samples (*n* = 3) were run on a LightCycler 480 thermal cycler (Roche) and analysed using the efficiency-corrected E-Method in Roche LightCycler 480 v1.5 software. Regional differences in *RAI1* expression levels were analysed by ANOVA, followed by post hoc *t* tests.

### Western blotting

Human hippocampal protein was extracted in 0.01 M phosphate buffer containing a protease inhibitor cocktail (Sigma) using mechanical homogenization and 3 freeze–thaw cycles. Homogenates were centrifuged for 10 min at 12,000 rpm at 4 °C. Total protein levels in each sample were quantified by the BCA assay (Pierce) and 50 μg total protein per lane was loaded onto a NuPAGE Novex Tris–acetate 3–8 % gradient mini-gel (Life Technologies). After separation, proteins were transferred onto a Hybond ECL nitrocellulose membrane (GE Healthcare) using a Mini Trans-Blot Cell (Bio-Rad) for 4 h and loading was checked with Ponceau S (Sigma). Membranes were probed with the primary antibody that was to be used for anti-RAI1 immunohistochemistry, rabbit anti-RAI1 (1:1000; Abcam). Labelled proteins were detected using a horseradish peroxidase (HRP)-conjugated anti-rabbit secondary antibody (Sigma; 1:5000) and enhanced chemiluminescence (ECL; Millipore), incubated for 5 min followed by exposure to X-ray film (Thermo Fisher Scientific).

### Subcellular fractionation

SH-SY5Y human neuroblastoma cells were grown in Dulbecco’s Modified Eagle (DMEM)/F-12 medium (Gibco), containing 10 % fetal calf serum and penicillin–streptomycin. Cells were harvested by trypsinization, pelleted by centrifugation and the medium discarded. Subcellular fractionation was carried out immediately using a protein and RNA isolation system kit (PARIS kit, Ambion), following the manufacturer’s protocol. Briefly, the cell pellet was resuspended in ice-cold PARIS cell fractionation buffer and incubated on ice for 10 min to lyse the cells. Nuclei were pelleted by centrifugation at 4 °C (500*g*, 5 min), and then the cytoplasmic fraction was removed into another tube and kept on ice. To reduce contamination by cytoplasmic proteins, the nuclear pellet was washed by gentle resuspension in cell fractionation buffer then centrifuged again. The supernatant was discarded and the nuclei were lysed in PARIS cell disruption buffer. A second cell pellet obtained at the same time was homogenized in ice-cold cell disruption buffer without fractionation to give a whole cell lysate. Anti-RAI1 western blotting was performed as described above. Western blotting against the nuclear protein histone H3 (rabbit anti-histone H3, 1:10.000; Abcam) was performed to confirm that the cytoplasmic fraction was free of nuclear contamination.

## Results

**Fig. 1:**
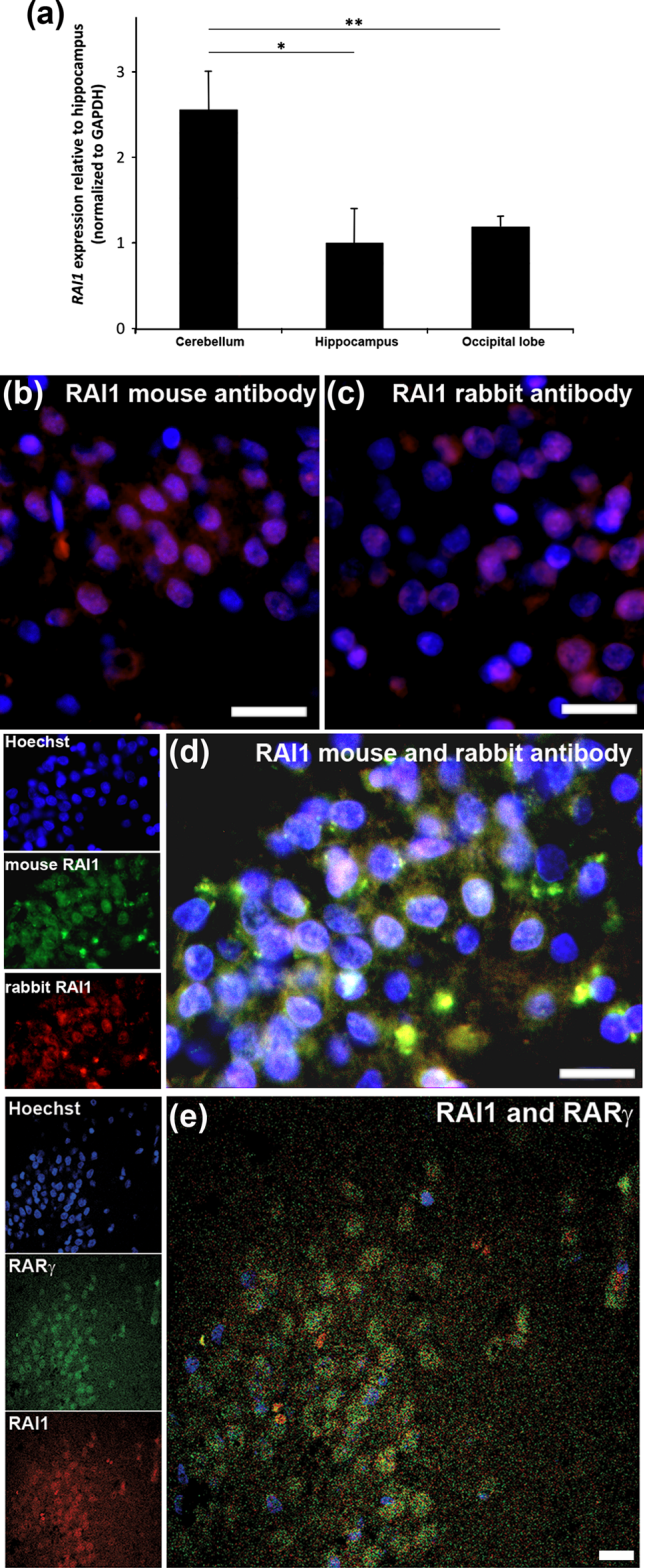
Expression of *RAI1* mRNA transcript in regions of the human brain and immunohistochemistry for RAI1 in the dentate gyrus. qPCR analysis of *RAI1* in the human cerebellum, hippocampus and occipital lobe of the cerebral cortex indicated its presence in all these regions (**a**). Cerebellar and occipital lobe expression is shown relative to hippocampal expression. Expression was significantly greater in the cerebellum compared to the hippocampus (*P* < 0.05; ANOVA followed by post hoc *t* test) and the occipital lobe (*P* < 0.01). Mouse (**b**) and rabbit (**c**) anti-RAI1 antibodies were compared and found to show similar strong expression in the human dentate gyrus and, when combined for double labelling, were found to be identical in distribution in both nucleus and cytoplasm in cells of the dentate gyrus, although expression varied between individual neurons (**d**). Confocal analysis of RAI1 expression also indicated expression of RAI1 in nuclei and cytoplasm, and expression in the same cells as RARγ (**e**). *Scale bars* 50 μm in **b**, **c**; 25 μm in **d**, **e**

The hippocampus and cerebellum are the regions of the mouse brain in which *RAI1* expression is strongest, reflecting its role in learning and motor function (Bi et al. 2007). qPCR was used to investigate regional expression of *RAI1* in the human brain. Figure 1a shows that the human cerebellum and hippocampus also show significant expression of *RAI1* transcript, together with the occipital lobe of the cortex. *RAI1* transcript levels were significantly higher in the cerebellum versus the hippocampus and occipital cortex. The distribution of the corresponding RAI1 protein has not previously been investigated in the human brain. To examine this, we employed an immunohistochemical method originally developed by Makitie et al., providing sensitive and specific staining with very little background autofluorescence (Makitie et al. 2010), which is very effective with the formalin-fixed sections used in this study (Fragoso et al. 2012). RAI1 distribution in the human hippocampus was first examined using two antibodies, each produced in a different species (rabbit and mouse) to allow double labelling with a variety of neuronal and glial markers. The distribution of labelling by these two antibodies in the dentate gyrus was very similar (Fig. 1b, c) and double labelling confirmed their staining pattern was identical (Fig. 1d). Such strong expression of RAI1 in the dentate gyrus is similar to what has been shown in the mouse hippocampus (Bi et al. 2007). RAI1 was apparently present in both nucleus and cytoplasm with the relative proportion in these compartments varying from cell to cell. Confocal imaging demonstrated that nuclear and cytoplasmic labelling was present in the same cell (Fig. 1e) and a comparison was also made with the retinoic acid receptor γ, which colocalized to the same cells.

**Fig. 2:**
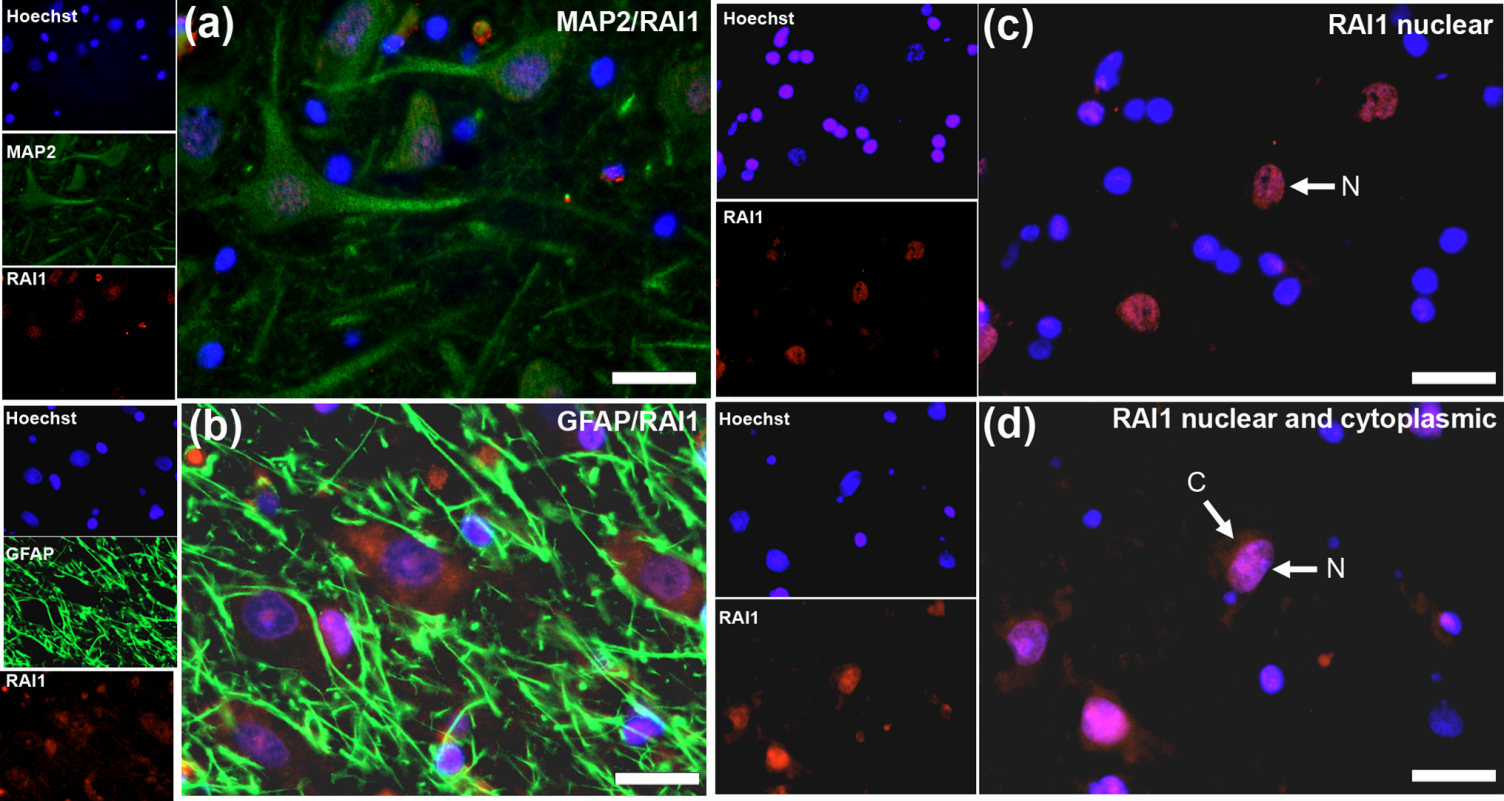
RAI1 expression in nucleus and cytoplasm of neurons in the hippocampal CA1 subfield. Double labelling of rabbit anti-RAI1 with MAP2 antibodies demonstrates expression of RAI1 in neurons of CA1 (**a**) while labelling with GFAP shows its absence in glia in this region (**b**). Of note was the differing subcellular distribution of RAI1—using the rabbit anti-RAI1 antibody, expression was confined to the nucleus in some cells (**c**), whereas RAI1 was present in both the nucleus and cytoplasm of others (**d**). *Scale bars* 25 μm

In the mouse, RAI1 is also expressed in the cornus ammonis (CA) of the hippocampus (Bi et al. 2007) and this was the case in human CA1 (Fig. 2a). Mouse RAI1 was described to be predominantly expressed in neurons (Bi et al. 2007) and this was investigated in CA1 of the human hippocampus by double labelling of RAI1 with MAP2 and GFAP, specific markers of neurons and glia respectively. RAI1 colocalised with MAP2, but not GFAP, demonstrating specific neuronal expression (Fig. 2a, b). However, it was noted that the subcellular localization of RAI1 was not the same in all neurons. As illustrated in Fig. 2, RAI1 expression in some neurons was almost exclusively nuclear (Fig. 2c), while in others it appeared to be present in both nucleus and cytoplasm (Fig. 2d).

**Fig. 3:**
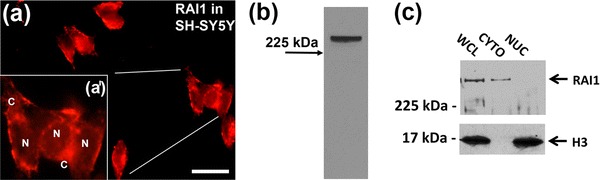
Expression of RAI1 in the cytoplasm of human SH-SY5Y cells. The expression of RAI1 in the cytoplasm was examined in a cell line in which RAI1 was apparently present in both the cytoplasm and nucleus (**a** and higher magnification view of three cells in **a** ^**ı**^). The rabbit anti-RAI1 detected a protein of approximately 280 kDa by western blotting (**b**). When SH-SY5Y cells were lysed and separated into nuclear and cytoplasmic fractions, the 280 kDa band was detected by western blotting in the whole cell (WCL) and cytosolic (CYTO) fraction but very little was present in the nuclear (NUC) fraction (**c**). The cytosolic fraction was uncontaminated by the nuclear fraction as indicated by the absence of the 17 kDa histone H3 protein (**c**). *Scale bar* in (**a**) 25 μm

This apparent cytoplasmic localization of RAI1 was surprising given that RAI1, when studied by induced expression in cell lines, showed only nuclear expression (Bi et al. 2005), and only truncated proteins lacking the nuclear localization signal were present in the cytoplasm (Carmona-Mora et al. 2010; Carmona-Mora et al. 2012). Anti-RAI1 immunohistochemistry, however, using two different antibodies showed apparent cytoplasmic and nuclear labelling in the hippocampus (Fig. 1b, c). To further examine whether endogenous RAI1 can be localized to the cytoplasm, we studied endogenous RAI1 in the SH-SY5Y human neuroblastoma cell, in which RAI1 expression has not previously been investigated. This cell line was chosen as a human neuroblastoma cell line and so showing some properties of human neural cells. In this cell line, RAI1 was predominantly found in the cytoplasm (Fig. 3a, a′). Western blotting was used to investigate RAI1 protein expression and a band was evident with a molecular weight similar to the approximately 260 kDa previously reported for RAI1 protein (Carmona-Mora et al. 2010) (Fig. 3b). To confirm that the cytoplasmic immunolabelling for RAI1 was an accurate reflection of the protein distribution in SH-SY5Y cells, nuclear and cytoplasmic fractions were separated using a PARIS kit (Ambion). The whole cell and cytoplasmic fractions were found to contain RAI1 and this fraction was demonstrated to be free of any nuclear contamination using antibodies to the nuclear protein histone H3 (Fig. 3c).

**Fig. 4:**
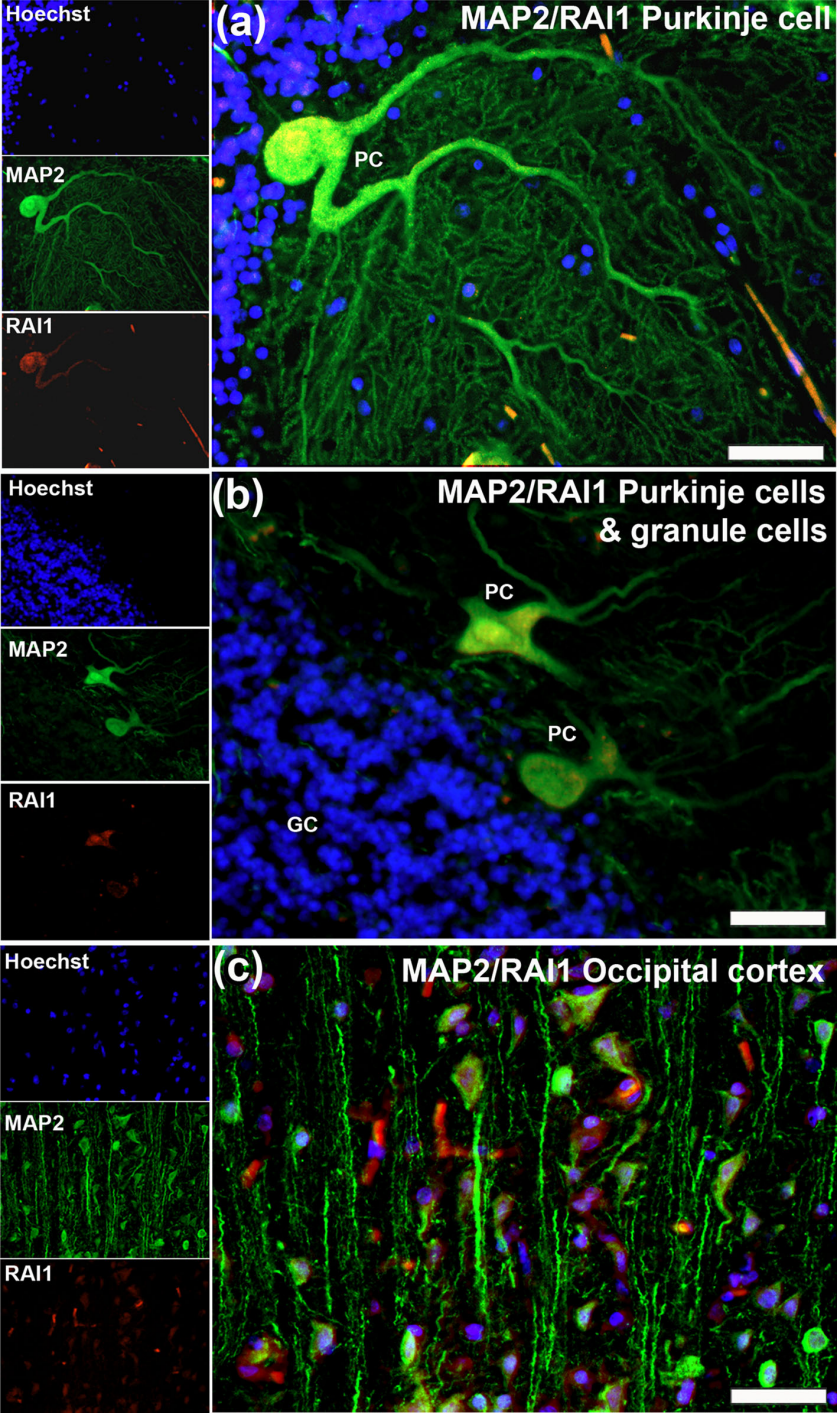
Expression of RAI1 in the human cerebellum and cerebral cortex. RAI1 was strongly expressed in cerebellar Purkinje cells (PC) and was observed in both the nucleus and the cytoplasm of the larger dendrites (**a, b**), but was absent from cerebellar granule neurons (GC in **b**). RAI1 was present in cells of layers II to IV of the occipital cortex (**c**). *Scale bars* 25 μm

Figure 1a showed the cerebellum and cerebral cortex to be regions of *RAI1* transcript expression. In the mouse cerebellum, *RAI1* is strongly expressed in the Purkinje cell layer (Bi et al. 2007). Similarly, RAI1 was very strongly expressed in human Purkinje cells (Fig. 4a), apparently in both the nucleus, the cell body and in the thicker dendritic branches. As in the mouse, RAI1 was absent from the cerebellar granule neurons (Fig. 4b). The low expression of MAP2 in the cerebellar granule neurons evident in Fig. 4b has previously been reported (Di Stefano et al. 2001). A second region of neuronal RAI1 expression was found to be the cerebral cortex and RAI1 protein was apparently present in both cytoplasm and nucleus of MAP2-positive neurons in the occipital cortex (Fig. 4c).

**Fig. 5:**
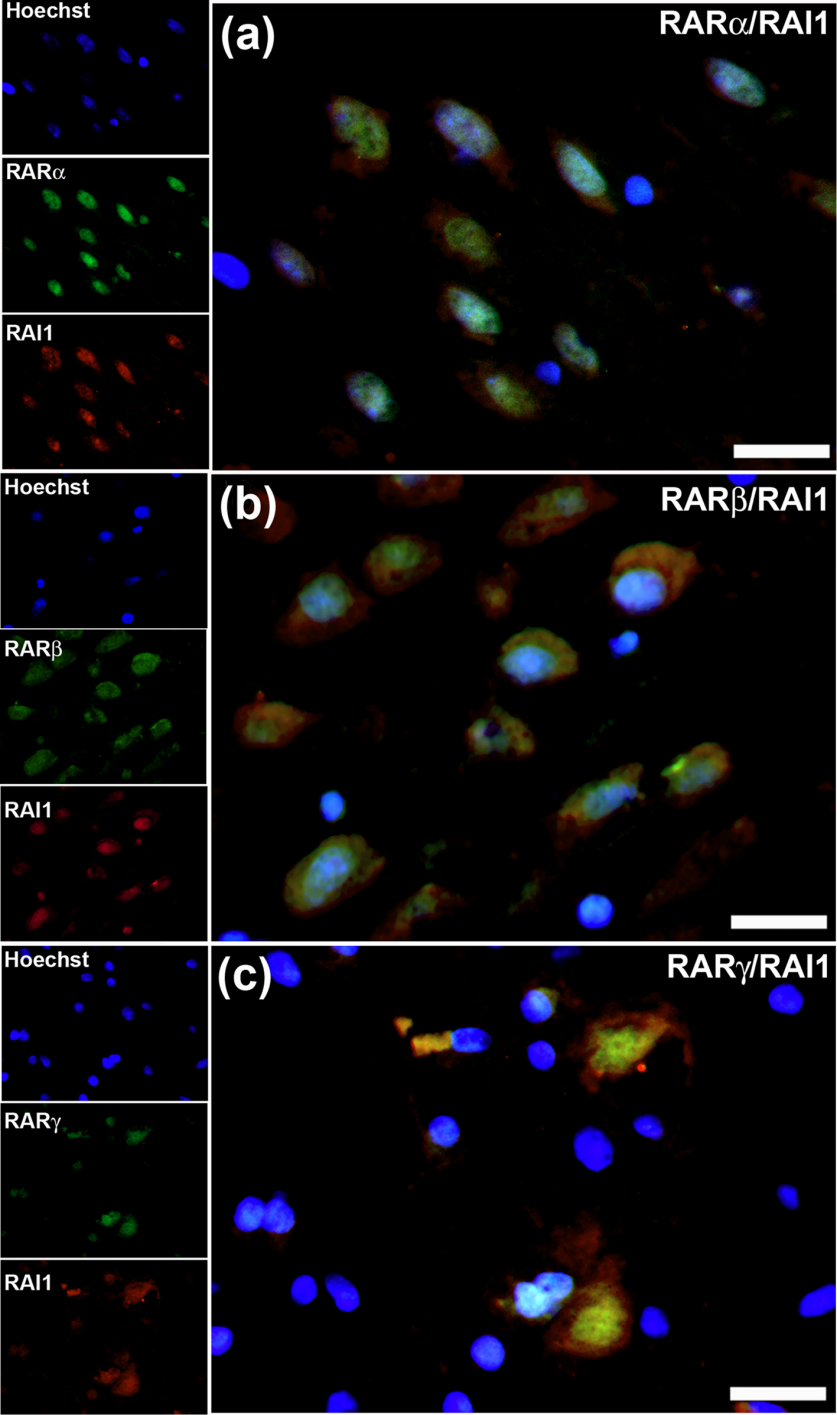
Colocalization of RAI1 with the retinoic acid receptors (RARs). In the hippocampal CA1 subfield, RAI1 was expressed in cells that also express RARα, which is predominantly in the nucleus (**a**), RARβ, present in both the nucleus and cytoplasm (**b**) and RARγ, which is mainly nuclear in expression (**c**). *Scale bars* 25 μm

RAI1 is named after its retinoic acid inducibility and, given our recent finding of strong expression of the retinoic acid receptors (RARs) in human hippocampal neurons (Fragoso et al. 2012), the relative distribution of RAI1 was compared with the three subclasses of these receptors. As previously found, RARα and RARγ were predominantly in the nucleus (Fig. 5a, c) and RARβ in both nucleus and cytoplasm (Fig. 5b), but most cells that express RAI1 also express the retinoic acid receptors (Fig. 5a–c).

## Discussion

Interest in *RAI1* has rapidly increased in the past few years because of its association with disease. Deletion of the chromosomal region 17p11.2 results in Smith–Magenis syndrome and haploinsufficiency of *RAI1* makes a major contribution to the resulting craniofacial abnormalities, behavioural problems and mental retardation. The reciprocal duplication of this region leads to Potocki–Lupski syndrome, which similarly results in mental retardation, but also hyperactivity and some autism-like features. This study shows, for the first time, the distribution of RAI1 protein in the adult human brain. RAI1 was found to be present in the majority of neurons in both hippocampus and cortex, as expected from earlier studies showing the human brain to be the tissue with highest *RAI1* expression and mRNA to be present in all brain regions examined except for the corpus callosum (Toulouse et al. 2003), similar to the distribution in the mouse (Imai et al. 1995). As found in the mouse (Bi et al. 2007), we found that RAI1 is absent from glial cells in the hippocampus and is strongly expressed in cerebellar Purkinje cells but not granule cells. The subcellular distribution of RAI1 was suggestive of expression of RAI1 in both the nucleus and cytoplasm.

The RAI1 protein has multiple putative nuclear localization sequences (NLS) (Slager et al. 2003; Carmona-Mora and Walz 2010) and when expressed in a cell line is transported into the nucleus (Carmona-Mora et al. 2010), while mutant forms of RAI1 lacking the NLS localize to the cytoplasm (Bi et al. 2005; Carmona-Mora et al. 2010; Carmona-Mora et al. 2012). Several studies points to RAI1 functioning as a transactivator (Seranski et al. 2001; Bi et al. 2004; Carmona-Mora et al. 2010) and it induces expression of the central circadian *circadian locomotor output cycles kaput* (CLOCK) gene while haploinsufficiency of RAI1 disrupts many circadian genes (Williams et al. 2012). RAI1’s regulation of *BDNF* in the hypothalamus may contribute to the hyperphagia and obesity seen in Smith–Magenis syndrome (Burns et al. 2010) and haploinsufficiency of *RAI1* results in abnormal expression of a number of genes associated with obesity, including *proopiomelanocortin* (*POMC*). Major gene changes in the hypothalamus in this condition also include alterations in growth hormone together with a number of homeobox-containing transcription factors (Burns et al. 2010).

These results point to RAI1’s presence in the nucleus to regulate transcription. When transfected into cell lines it localizes to the nucleus and is associated with chromatin (Bi et al. 2005, 2010; Carmona-Mora et al. 2012) while a study of lymphoblastoid cells from normal or Smith–Magenis patients indicated expression in the nucleus (Carmona-Mora et al. 2012). Although the smallest predicted splicing variant of RAI1 lacks the NLS and localizes to the cytoplasm (Burns et al. 2010), this is not the large form of RAI1 seen by western blotting in this study. However, it is possible that the protein conformation of this smaller form is such that it is only recognized by antibody in fixed cells and not once denatured for gel electrophoresis and western blotting. It is of interest though that the original identification of the endogenous *RAI1* gene, in the mouse P19 embryonic carcinoma cell line (Imai et al. 1995), also described the protein to be present in the cytoplasm of neurons of the adult mouse brain and thus a cytoplasmic role was proposed. It is possible that this is also the smaller form of RAI1. This present study suggests that forms of endogenous RAI1 in the human brain can be expressed in both the nucleus and cytoplasm in neurons suggesting a role for this cytoplasmic expression, even if just to inhibit RAI1’s nuclear action to regulate transcription. The relative intensity of expression of RAI1 in the nucleus and cytoplasm differed even between neurons of the same type implying a variance between these cells that may reflect, for instance, functional activity.

The inducibility by retinoic acid that gives RAI1 its name was reflected in the parallels in this study between the neuronal expression of RAI1 and overlap with retinoic acid receptor expression. Retinoic acid can regulate *RAI1* (Imai et al. 1995) and there is a retinoic acid response element (RARE) just upstream of exon 1 (Toulouse et al. 2003); an analysis of the *RAI1* promoter found three DR2-type RAREs which are bound by retinoic acid receptors as determined by chromatin immunoprecipitation assay (Laperriere et al. 2007). In the contrary direction, there may be some element of retinoid signalling downstream of RAI1 action. Cell line analysis of genes altered by haploinsufficiency of *RAI1* found *retinoid x receptor beta* (*RXRB*) as one of the ten main genes up-regulated and also proposed an interrelatedness in phenotype with several diseases that included DiGeorge syndrome and fragile X syndrome (Girirajan et al. 2009). Both of these developmental disorders have been associated with abnormal retinoic acid signalling (Guris et al. 2006; Soden and Chen 2010). The interrelationship between retinoic acid, its receptors (RARs) and RAI1 are not yet understood. Given that RAI1 promotes transcription (Carmona-Mora et al. 2012) and may act as transcriptional co-activator like its homologue TCF20 [also called SPBP (Darvekar et al. 2012)], perhaps RAI1 and the RARs themselves interact. The action of nuclear receptors, such as the androgen and oestrogen receptors, as well as the RARs, are tightly controlled by co-activators and co-repressors (Rochette-Egly and Germain 2009). It is of note that TCF20 acts as a co-activator for the androgen receptor (Elvenes et al. 2011) but is a repressor of phosphorylated estrogen receptor (Gburcik et al. 2005).

The findings of this report provide the first demonstration of high expression of the essential protein RAI1 in neurons of several regions of the human brain. Its presence in both nucleus and cytoplasm may indicate shuttling, and so control, of this protein whose function includes regulation of transcription. Alternatively, different splice variants may be localized to the two subcellular compartments. The coexpression of RAI1 with the retinoic acid receptors supports a functional relationship between these two transcription regulatory proteins.
